# Functional Specialization of Clathrin‐Independent Endocytic Routes: Mechanisms and Cellular Roles

**DOI:** 10.1111/tra.70049

**Published:** 2026-08-13

**Authors:** Andrea Francesco Benvenuto, Amir Fardin, Matteo Balestra, Pier Paolo Di Fiore, Chiara Tordonato, Sara Sigismund

**Affiliations:** ^1^ IEO, European Institute of Oncology IRCCS Milan Italy; ^2^ Department of Oncology and Hematology‐Oncology Università Degli Studi di Milano Milan Italy

## Abstract

Clathrin‐independent endocytosis (CIE) comprises a diverse repertoire of internalization mechanisms that operate in parallel with clathrin‐mediated endocytosis. While these mechanisms share certain characteristics, they are governed by distinct molecular principles and regulatory logic. In this review, we provide an overview of CIE mechanisms, with a particular focus on the functional specialization of individual routes. We trace the historical emergence of CIE concepts and highlight how bacterial toxins and viruses have served as pivotal discovery tools, revealing lipid‐driven and lectin‐mediated mechanisms of membrane bending and internalization. Furthermore, we delineate the molecular frameworks of major CIE mechanisms and discuss experimental strategies for their dissection. We emphasize how the selection of a specific endocytic route dictates cargo fate, directing receptors and adhesion molecules toward recycling, retrograde trafficking, or degradation to modulate signaling amplitude, polarity, migration and epithelial barrier functions. Finally, we offer an evolutionary perspective on “coatless” CIE‐like mechanisms.

## Graphical abstract

Distinct clathrin‐independent endocytic (CIE) routes support specialized cellular functions across different contexts. Created with Biorender.com.

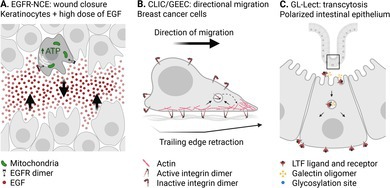

## 1 Introduction

Endocytosis is a fundamental cellular process that governs many facets of physiology, development, and disease. Beyond its canonical role in nutrient uptake and membrane homeostasis, endocytosis is now recognized as a master organizer of signaling, cell polarity, migration, and tissue homeostasis. By integrating extensively with signaling networks, endocytic trafficking shapes the magnitude and duration of receptor‐mediated signaling events. From an evolutionary standpoint, endocytosis likely emerged as a primordial mechanism for the internalization of extracellular fluids, nutrients, and macromolecules. This ancestral function for uptake and intracellular processing was likely foundational for early eukaryotic life and may have facilitated endosymbiotic events, most notably those leading to the acquisition of mitochondria and plastids [1].

Clathrin‐mediated endocytosis (CME) is the best‐studied endocytic pathway and has long served as the primary model for membrane internalization. It is characterized by distinctive morphological and molecular features that define a highly orchestrated process involving cargo selection, membrane bending, vesicle scission, and uncoating [2, 3, 4, 5, 6, 7, 8]. CME operates both constitutively (e.g., transferrin uptake) and in a ligand‐induced manner (e.g., growth factor receptor internalization) [9]. These events exhibit rapid turnover, typically occurring within 30–60 s, and are subject to stringent quality‐control mechanisms in which specialized adaptor proteins ensure cargo thresholds are met before vesicle maturation [10, 11, 12]. For detailed molecular and functional insights into CME, readers are referred to a recent comprehensive review [13] as well as contributions in this issue.

The discovery and characterization of clathrin‐independent endocytosis (CIE) pathways have expanded the conceptual framework of endocytosis established through decades of work on CME. CIE encompasses a heterogenous collection of uptake mechanisms that lack the stereotypical morphological hallmarks of CME and exhibit considerable variation across cell types and physiological contexts [14, 15]. These mechanisms diverge in their molecular machinery, kinetics, and regulatory logic, underscoring the diversity of cellular strategies available for membrane internalization. This diversity, compounded by the absence of a universal coat structure analogous to clathrin, has historically impeded their identification, classification, and mechanistic dissection. Consequently, progress in the CIE field has been more fragmented and less linear than the cohesive advancement seen in that of CME. Nevertheless, insights gained from CIE have complemented the classical CME paradigm by revealing additional layers of mechanistic and functional specialization within the endocytic system.

In this review, we provide an integrated synthesis of CIE, prioritizing the functional specialization of its distinct mechanisms. We first examine the historical emergence of CIE concepts, contextualizing key discoveries relative to the established CME framework. We then discuss how bacterial toxins and viruses have served as powerful tools to uncover and define specific CIE routes. Subsequently, we summarize the current state‐of‐the‐art regarding CIE mechanisms, focusing on their molecular players and regulatory principles, and analyze how integrated genetic and chemical approaches clarify these processes. We further examine the repertoire of cargoes internalized via CIE with emphasis on adhesion molecules, and discuss the functional implications of these internalization mechanisms in physiology and cancer. Finally, we conclude by offering an evolutionary perspective on CIE, positing its role as an ancient and adaptable mode of cellular uptake.

### 1.1 A Brief History of CIE

The concept that cells actively internalize material from their surroundings emerged from the foundational work of Ilya Metschnikoff, who in 1883 defined *phagocytosis* to describe the ingestion and degradation of particulate material by specialized cells [16, 17]. Derived from the Greek *phagein* (“to eat”) and *kytos* (“container”), the term captured the concept of the cell (the “container”) as an active participant in environmental sampling. Subsequent advancements in microscopy during the early 20th century revealed that cells also internalize extracellular fluid via membrane‐bound “vacuoles.” In 1931, Warren H. Lewis termed the process *pinocytosis*, or “cell drinking” [18], which was soon recognized as a fundamental homeostatic mechanism for nutrient acquisition and membrane turnover.

In 1953, George E. Palade described small, uniformly sized and shaped invaginations of the plasma membrane (PM) in heart endothelial cells, which he termed *plasmalemmal vesicles* [19]. Shortly thereafter, Eichi Yamada reported similar flask‐shaped structures at the basolateral surface of gallbladder epithelial cells and coined the term “*caveolae intracellulares*” based on their resemblance to small caves [20]. Although caveolae were originally proposed to function as endocytic carriers, they are now primarily viewed as mechanosensitive membrane domains that buffer membrane tension [21, 22]. Their endocytic activity is considered conditional rather than constitutive, and appears to be restricted to specific cellular contexts, such as endothelial cells. As caveolae constitute a distinct and highly specialized area of research, they will not be discussed further here; instead, we refer the reader to recent comprehensive reviews on the subject [23, 24, 25].

A major conceptual advance came in 1964, when Roth and Porter described a specialized vesicle‐mediated uptake mechanism in mosquito oocytes involving PM pits coated with “bristle‐like” structures that invaginated and pinched off to internalize yolk proteins [26]. These “bristle‐like” structures were later identified as clathrin lattices following Barbara Pearse's successful purification of clathrin‐coated vesicles in 1975 [27]. The subsequent identification of the AP‐2 adaptor complex established the molecular basis for cargo selection and clathrin recruitment [2, 6, 28], positioning CME as the dominant paradigm in membrane trafficking for decades.

Concurrently, Ralph M. Steinman and colleagues performed seminal quantitative studies of fluid‐phase endocytosis using horseradish peroxidase (HRP) as a tracer [29, 30, 31]. By integrating kinetic measurements with stereological analysis, they provided the first rigorous quantification of membrane flux and established HRP as the gold‐standard marker for constitutive bulk uptake.

The first specific evidence for CIE emerged in 1978, with the observation of HRP internalization via smooth, ring‐like vesicles—now recognized as CLIC/GEEC (clathrin‐independent carriers/glycosylphosphatidylinositol (GPI)‐anchored‐protein‐enriched early endosomal compartments) carriers [32, 33]. This was followed by the discovery that MHC class I molecules could undergo antibody‐induced internalization via non‐clathrin‐coated vesicles [34]. A critical distinction was further established in 1985, when Maryse Moya et al. demonstrated that while CME inhibition blocked diphtheria toxin entry, ricin continued to be internalized efficiently, confirming the existence of alternative, clathrin‐independent routes [35].

By the 1990s and early 2000s, the field began to delineate specific CIE mechanisms. Lund et al. identified a high‐capacity, low‐affinity route for EGFR internalization activated at saturating ligand concentrations [36]. Subsequently, Lamaze et al. characterized the IL‐2 receptor uptake mechanism, noting its dependence on detergent‐resistant domains, dynamin, and Rho‐family GTPases [37]. This mechanism was later termed fast endophilin‐mediated endocytosis (FEME) and shown to mediate the uptake of different RTKs and G protein–coupled receptors (GPCRs), particularly at the leading edge of migrating cells [38]. Similarly, our group identified EGFR‐non‐clathrin endocytosis (EGFR‐NCE), a mechanism triggered by high EGF doses that specifically requires receptor ubiquitination [39].

In recent years, the identification and characterization of CIE mechanisms have accelerated, propelled by high resolution imaging technologies including super‐resolution fluorescence microscopy, correlative light‐electron microscopy (CLEM), cryo‐electron tomography (cryo‐ET), lattice light‐sheet microscopy (LLSM), and conventional transmission electron microscopy (TEM). These tools have moved the field beyond simple morphological descriptions, providing a multidimensional understanding of CIE at the molecular and functional levels [37, 40, 41, 42, 43, 44].

### 1.2 Toxins and Viruses as Discovery Tools for CIE

Much of our understanding of CIE has arisen from studies of bacterial toxins and viruses, most notably cholera toxin (CTx), shiga toxin (STx), ricin, and the polyomavirus, simian virus 40 (SV40) [35, 45, 46, 47, 48]. These studies revealed that cells retain robust endocytic activity even when CME is inhibited [49, 50, 51, 52], thereby establishing the existence of alternative internalization routes. It subsequently became clear that many of these pathogens can exploit either CME or CIE mechanisms, depending on the pathogen species, host cell type, and experimental context. As a result, toxins and viruses have served as powerful experimental tools for dissecting endocytic mechanisms. Moreover, since pathogen uptake constitutes the defining early step of infection, the route of entry has major implications for host range, tissue tropism, and disease emergence [45, 48].

One of the earliest lines of evidence for pathogen exploitation of CIE emerged in the 1980s, when the internalization of ricin was shown to persist under acidic conditions and intracellular potassium depletion—conditions that block clathrin‐mediated uptake of the prototypical cargo, the transferrin receptor [35, 49]. Toxin studies were particularly instrumental in demonstrating that lipid‐binding pathogens can drive uptake independently of clathrin, caveolin, and in some cases dynamin, while relying on the actin–microtubule cytoskeleton [50, 51, 53]. In particular, work on CTx and STx established the central role of glycosphingolipids and multivalent lipid engagement in membrane bending, leading to the recognition that cargo geometry and membrane composition can themselves initiate endocytosis [54, 55, 56, 57]. These findings culminated in the formulation of the glycolipid–lectin (GL‐Lect) hypothesis [58, 59, 60], which proposes that CIE can be initiated by the multivalent binding of lectins to glycosphingolipids at the PM, without the need for canonical protein coats. According to this model, lectins, such as the B subunits of STx or CTx, cluster their glycolipid receptors (e.g., Gb3 or GM1), reorganizing lipids and generating membrane curvature through collective, geometry‐driven interactions. When sufficiently amplified, this lipid‐cargo coupling is sufficient to drive membrane invagination, scission and internalization, often in association with actin remodeling and downstream trafficking machinery. The GL‐Lect hypothesis thus unified previously disparate CIE routes by highlighting lipid‐driven membrane remodeling as a core organizing principle.

In parallel, viruses have profoundly shaped the field by acting as selective reporters of endocytic pathways. Early evidence for viral entry via CIE dates to 1996, when SV40 was shown to be internalized via a clathrin‐independent, caveolae‐dependent route [48], later shown to require cholesterol, actin, and microtubules [48, 61, 62, 63]. Subsequent work revealed that SV40 can also enter cells through caveolae‐independent CIE mechanisms that nonetheless depend on cholesterol‐enriched PM domains, often referred to as “lipid rafts” [63]. In 2005, Lucas Pelkmans et al. extended these findings with a systematic kinome‐wide RNAi screen that exploited viruses as pathway‐specific endocytic reporters; SV40 served as a readout for caveolae/raft‐dependent CIE, whereas vesicular stomatitis virus (VSV) was used to monitor CME. By quantifying the effects of individual kinase knockdowns on SV40 and VSV infection, alongside transferrin uptake (CME‐specific cargo), the study defined route‐specific signaling “signatures” and identified kinases that differentially regulate CME and CIE, thereby providing a systems‐level framework for dissecting endocytic signaling networks [64].

Subsequent studies leveraged viral entry to uncover additional CIE mechanisms exploited by clinically important pathogens, including filoviruses, polyomaviruses, influenza viruses, and adenoviruses [65, 66, 67, 68, 69, 70]. Notably, a recent study demonstrated that CLIC/GEEC endocytosis mediates the uptake of HIV‐1 virus‐like particles in macrophages, leading to the formation of virus‐containing compartments that support viral persistence [71]. Viruses have also provided safe surrogate systems—such as pseudotyped VSV particles—to dissect the entry mechanisms of highly pathogenic agents, including Ebola virus and SARS‐CoV‐2. These approaches enabled the rapid and safe identification of host trafficking nodes essential for infection [72, 73, 74]. Collectively, studies of toxins and viruses have not only established the existence and diversity of CIE mechanisms but also underscored their physiological and pathological significance alongside CME.

### 1.3 Mechanisms of Clathrin‐Independent Endocytosis

**FIGURE 1:**
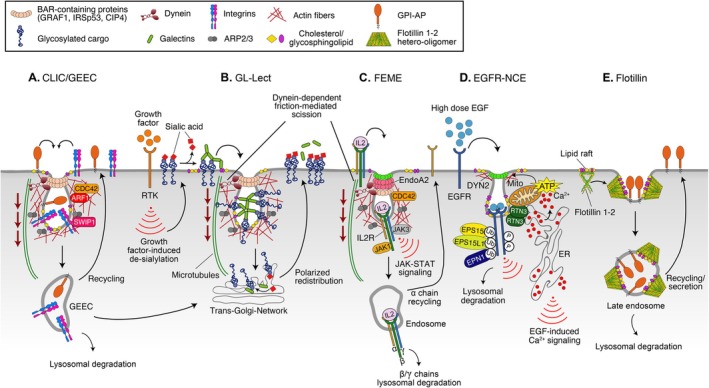
Mechanisms of clathrin‐independent endocytosis. (A) CLIC/GEEC (Clathrin‐Independent Carriers/GPI‐Enriched Endosomal Compartments) is a constitutively active mechanism that internalizes GPI‐anchored proteins (GPI‐APs), glycoproteins (e.g., integrins) and glycosphingolipids. CLIC formation and elongation are driven by BAR‐domain‐containing proteins (IRSp53, CIP4), small GTPases (CDC42, ARF1) and the Actin nucleator ARP2/3. Swiprosin‐1 (SWIP1) acts as specific adaptor coupling active β1‐integrin with the CLIC machinery. Dynein‐dependent, friction‐mediated scission releases CLICs whose fate depends on the cellular context and the cargo; they can be targeted to the trans‐Golgi network, recycled to the plasma membrane (PM) or sent to lysosomes for degradation. (B) GL‐Lect (glycolipid‐lectin) endocytosis is responsible for the internalization of several glycosylated cargoes and toxins. Mechanistically, it is driven by galectin oligomerization and binding to glycosylated cargoes and glycosphingolipids, inducing PM nanodomain assembly and curvature. Efficient galectin binding is promoted by cargo desialylation, which is triggered by receptor tyrosine kinase (RTK) signaling upon growth factor stimulation (termed “desialylation glycoswitch”). Tubule elongation is mediated by the action of Actin and dynein, while the subsequent scission is generally dynamin‐independent and occurs via a friction‐mediated mechanism. After scission, CLICs follow the retrograde trafficking route to the trans‐Golgi network where cargoes are resialylated before being recycled to defined regions of the PM, allowing polarized redistribution of cargoes. (C) FEME (Fast Endophilin Mediated Endocytosis) is activated by ligand stimulation and mediates the internalization of several receptors, including IL‐2R. Upon ligand‐receptor binding, CDC42 recruits BAR‐containing proteins (e.g., CIP4), which act as scaffolds for endophilin A2 (EndoA2). At these sites, EndoA2 oligomerization promotes membrane bending and tubulo‐vesicular carrier formation. Carrier scission is determined by the coordinated action of dynamin 2 (DYN2), Actin and EndoA2 in a friction‐driven scission mechanism. Although FEME leads to recycling/degradation of certain cargoes, for example, IL‐2R as represented here (where IL2Rβ/γ chains are targeted for degradation, and the α chain is recycled), the fate of other cargoes remains unknown. (D) EGFR‐NCE (EGFR‐non‐clathrin‐endocytosis) is specifically activated only upon high‐dose EGF stimulation and mediates the internalization of EGFR and glycosylated PM receptors (e.g., CD147, not shown). After stimulation, the EGFR is ubiquitinated and recognized by ubiquitin‐binding domain‐containing proteins (EPS15, EPS15L1, EPN1), which direct receptors toward EGFR‐NCE. Membrane bending and tubule elongation are sustained by a molecular platform based on tripartite contact sites between the PM, the endoplasmic reticulum (ER) and mitochondria, which are dependent on RTN3. At these sites, the ER releases Ca^2+^, which is sensed by mitochondria to produce the ATP necessary for cortical Actin remodeling. Actin and DYN2 cooperate in vesicle scission. Cargoes are then destined for lysosomal degradation. (E) Flotillin‐mediated endocytosis is responsible for the internalization of GPI‐APs and RTKs. Flotillin 1 and 2 form hetero‐oligomers in sphingolipid‐enriched microdomains (lipid rafts) of the PM. These oligomers promote membrane curvature, clustering and subsequent internalization of the cargoes, which are then directed back to the PM for recycling/secretion or to lysosomes for degradation.

While CIE mechanisms share similar morphological features and common characteristics, such as clathrin independence, stimulus‐dependent regulation, and coupling to actin dynamics (Figure 1), they rely on distinct molecular modules to confer cargo selectivity, drive membrane remodeling, and generate context‐specific trafficking outcomes. This versatility enables cells to adapt membrane internalization, signaling, and metabolic responses across physiological and pathological settings. In this section, we provide a concise overview of the molecular basis of the best‐characterized CIE mechanisms. For more detailed mechanistic insights, we refer readers to recent reviews [75, 76].

#### 1.3.1 Clathrin‐Independent Carriers/GPI‐Enriched Endosomal Compartments (CLIC/GEEC)

CLIC/GEEC (Figure 1A) is a constitutively active mechanism that internalizes GPI‐anchored proteins (e.g., CD59, CD55, Thy‐1, folate receptor), glycosphingolipids (e.g., GM1 bound by cholera toxin B‐subunit, CTxB), and certain glycoproteins (e.g., CD44, CD147, active α₅β₁ integrin). Uptake occurs through tubular PM invaginations (~120 nm diameter, 100–200 nm in length) termed CLICs, which mature into elongated, tubulo‐vesicular early endosomal compartments (GEECs). CLIC/GEEC operates prominently at the leading edge of polarized migrating cells such as fibroblasts and epithelial cells, as well as in apico–basally polarized epithelia, where it mediates apical uptake [77, 78].

Rather than representing a single pathway, CLIC/GEEC encompasses a family of closely related mechanisms that differ in their specific adaptors and regulators yet converge on similar morphologies and cargo profiles. Core regulators include: (i) BAR‐domain–containing proteins, such as IRSp53, endophilins, and CIP4; (ii) small GTPases, including CDC42 and ARF1 together with its GEF GBF1 [78, 79]; (iii) the Rho‐GAP‐domain–containing protein GRAF1; and (iv) the actin nucleator ARP2/3 [15]. Together, these factors coordinate tubular elongation and CLIC formation, with membrane scission occurring predominantly through dynamin‐independent mechanisms that require actin polymerization and dynein‐mediated transport, although a dynamin contribution has been reported in certain contexts [80]. Recently, the actin‐binding protein Swip1 was identified as a cargo‐specific adaptor that couples active conformers of β1‐integrin to components of the CLIC machinery [81]. CLICs deliver cargo to RAB5‐negative GEECs, which subsequently mature into Rab5‐positive sorting endosomes. From there, cargo can be targeted for lysosomal degradation, recycled back to the PM, or transported via retrograde pathways to the trans‐Golgi network, as in the case of toxins and integrins [82].

Conceptually, CLIC/GEEC can be viewed as a prototypical, coatless CIE mechanism defined by a conserved molecular core, with other CIE pathways representing specialized variants. For example, GL‐Lect‐driven endocytosis shares several features with CLIC/GEEC, including: (i) The morphology of tubular PM invaginations and CLIC‐like carriers; (ii) a scission mechanism that is generally dynamin‐independent and relies on actin polymerization together with dynein‐driven pulling forces along microtubules (friction‐mediated scission) [80]; and (iii) the participation of membrane curvature‐generating factors, including BAR‐domain‐containing proteins, although the specific BAR proteins involved differ between the two pathways. However, GL‐Lect‐driven endocytosis differs in the mechanisms that initiate cargo recruitment and membrane bending, relying on galectin oligomerization and glycoprotein‐glycosphingolipid interactions to drive nanodomain assembly, membrane curvature, and invagination (see more details in the next subsection).

#### 1.3.2 Glycolipid‐Lectin (GL‐Lect) Driven Endocytosis

GL‐Lect driven endocytosis (Figure 1B) internalizes diverse cargoes, including integrins, toxins, CD44, and CD166, across multiple cell types, such as breast cancer cells, fibroblasts, retinal pigment epithelial cells, and polarized epithelia including the mouse intestine in response to galectin binding [83]; it can also be triggered by growth factors (see below). The mechanism couples galectin binding and oligomerization of glycosylated cargo with glycosphingolipid clustering at the PM, resulting in membrane curvature, tubular invagination, and uptake.

GL‐Lect driven endocytosis was originally defined for galectin‐3 but has also been suggested to involve other galectins, such as galectin‐8 [84]. Galectins are a family of β‐galactoside–binding proteins that are secreted via non‐classical pathways and bind glycans on membrane glycoproteins. During GL‐Lect uptake, extracellular galectin‐3 is recruited to the PM as a monomer and subsequently oligomerizes into tetramers. These oligomers multivalently engage glycosylated cargo and glycosphingolipids, cross‐linking membrane components to drive nanodomain assembly, membrane bending, and the formation of tubular endocytic pits from which CLICs emerge [83]. Spatial reorganization of N‐glycans on cargo is critical for efficient galectin‐3 recruitment, a process termed the “conformational glycoswitch” [85]. This has been demonstrated for α₅β₁ integrin, in which the bent‐closed conformation presents N‐glycan arrangements that more efficiently nucleate galectin‐3 oligomers than the extended, active state [86]. GL‐Lect driven endocytosis can also be regulated by growth factors through a related “desialylation glycoswitch” mechanism [85] (see Section 5). Bacterial toxins such as STx and CTx hijack this machinery through their B‐subunits, which directly cross‐link membrane‐embedded glycosphingolipids (Gb₃ and GM₁, respectively), promoting lipid domain clustering and curvature generation that trigger GL‐Lect driven internalization [58, 83, 85, 86, 87].

Scission of GL‐Lect‐derived CLICs is typically mediated through actin‐driven, dynamin‐independent mechanisms, although dynamin involvement has been reported under specific cellular or cargo‐dependent conditions [58]. Following release, CLICs undergo retrograde transport to the Golgi apparatus and are subsequently recycled in a polarized manner to defined regions of the PM. This targeted trafficking enables redistribution of cargoes, such as integrins and adhesion molecules, to support cell polarity, directional migration, and localized signaling, while also shaping the routes exploited by toxins and viruses. Thus, GL‐Lect‐driven endocytosis illustrates how glycan‐dependent lectin oligomerization and lipid clustering generate coatless, cargo‐selective carriers, highlighting the versatility and physiological relevance of CIE mechanisms.

#### 1.3.3 Fast Endophilin‐Mediated Endocytosis (FEME)

FEME (Figure 1C) is a rapid, dynamin‐dependent CIE mechanism responsible for the ligand‐induced internalization of GPCRs, RTKs, some dopaminergic, adrenergic and muscarinic receptors, as well as the IL‐2R [38]. It is typically active in retinal pigment epithelial cells, fibroblasts and endothelial cells (e.g., HUVEC) [38, 88, 89, 90]. FEME is initiated upon ligand stimulation, which activates receptors and CDC42 signaling at discrete PM regions. Activated CDC42 recruits F‐BAR proteins (FBP17, CIP4), which assemble into small membrane patches that serve as scaffolds for endophilin A2. Endophilin A2 is then recruited to these patches and oligomerizes via its N‐BAR domain, amplifying membrane curvature and driving rapid formation of tubulo‐vesicular carriers [38]. FEME tubules display substantial morphological heterogeneity, ranging from 100 nm to several μm in length. Carrier scission is mediated by the coordinated action of dynamin, endophilin A2 and actin through a friction‐driven mechanism [80, 90]. Following scission, FEME carriers are transported along microtubules by the motor protein dynein [91] until fusion with early endosomes and subsequent trafficking to late endosomes and lysosomes [88].

Overall, FEME shares common features with CLIC/GEEC and GL‐Lect driven endocytosis such as rapid carrier formation, actin involvement, and the generation of uncoated, tubular endocytic intermediates while differing in cargo specificity, triggers, and regulatory modules.

#### 1.3.4 EGFR–Non‐Clathrin Endocytosis (EGFR‐NCE)

EGFR‐NCE (Figure 1D) is activated in epithelial cells by high, saturating concentrations of EGF (> 3–10 ng/mL) [39] and is characterized by rapid internalization through tubular PM invaginations morphologically similar to those observed in CLIC/GEEC and GL‐Lect endocytosis [43]. In addition to EGFR, other receptors, including CD147/basigin and HGFR, can utilize this pathway [43, 44].

A distinctive feature of EGFR‐NCE is its dependence on tripartite contact sites between the PM, endoplasmic reticulum (ER), and mitochondria, in a process dependent on the ER‐resident protein reticulon‐3 (RTN3) [43, 44]. These platforms coordinate membrane remodeling and dynamin‐dependent scission through localized ER Ca^2+^ release and enhanced mitochondrial ATP production [44]. EGFR‐NCE further requires CBL‐mediated receptor ubiquitination, which promotes the recruitment of ubiquitin‐binding adaptors such as EPS15, EPS15L1, and EPN1, directing EGFR towards lysosomal degradation [39, 92]. Thus, EGFR‐NCE functions as a key mechanism for receptor downregulation under conditions of intense stimulation. However, how receptor ubiquitination and ubiquitin‐binding adaptors mechanistically coordinate NCE and tripartite contact site formation remains unresolved. Moreover, since these adaptors also function in CME [39, 93], the basis for their pathway‐specific regulation remains an open question.

#### 1.3.5 Flotillin‐Mediated Endocytosis

Flotillin 1 and flotillin 2 are highly conserved, ubiquitously expressed members of the SPFH (stomatin, prohibitin, flotillin, HflK/C) superfamily that assemble into hetero‐oligomeric scaffolds at the cytoplasmic face of the PM within sphingolipid‐enriched, caveolin‐independent microdomains. These microdomains function as platforms for cargo clustering and signaling, promoting the selective concentration of specific transmembrane proteins [94, 95]. Consistent with this role, flotillin‐mediated endocytosis (Figure 1E) is closely linked to PM remodeling and highlights the function of flotillin scaffolds as multifunctional membrane‐organizing platforms. In various epithelial, neuronal, and hematopoietic cell types, flotillin‐enriched PM domains define a CIE route that generates small, uncoated flotillin‐positive vesicles, which subsequently fuse with early endosomes [96, 97]. This pathway is regulated by Fyn kinase, underscoring the importance of signaling‐dependent control in flotillin trafficking [96]. Through their ability to organize membrane microdomains, promote membrane curvature, and facilitate vesicle budding, flotillin complexes drive the clustering and internalization of diverse cargoes, including RTKs, adhesion molecules (e.g., cadherins and GPI‐anchored proteins), and various neurotransmitter transporters and receptors [98, 99, 100, 101]. After internalization, cargoes can be directed back to the PM for recycling or to lysosomes for degradation depending on the context [102, 103, 104]. This scaffold‐based organization is further linked to pathological trafficking programs, such as the “upregulated flotillin‐induced trafficking” (UFIT) [100, 101]. Collectively, these findings identify flotillin scaffolds as central regulators of raft‐associated trafficking and signaling, with broad implications for membrane dynamics and cellular behavior. Accordingly, flotillin‐mediated endocytosis represents a raft‐based variant of CIE that illustrates the importance of PM microdomain organization in cargo selection and internalization.

#### 1.3.6 Additional Clathrin‐Independent Mechanisms

Several additional CIE mechanisms have been described, particularly in neuronal cells. These include:

#### 1.3.7 ADBE (Activity‐Dependent Bulk Endocytosis)

ADBE is a rapid, high‐capacity, activity‐dependent CIE mechanism that operates at neuronal synapses and is triggered by elevated intracellular Ca^2+^ levels. Its initiation requires the Ca^2+^‐dependent phosphatase calcineurin, which coordinates the dephosphorylation of endocytic components [105]. ADBE is further regulated by the kinases Cdk5 and GSK3β, which control the phosphorylation state of key effectors, including the F‐BAR protein syndapin‐I, a mediator of membrane bending during ADBE. Together with dynamin‐1 and actin polymerization, syndapin‐I promotes membrane scission and the formation of large macropinocytic‐like endosomes (> 500 nm in diameter) [105, 106]. Functionally, ADBE mediates the retrieval of synaptic membrane and associated proteins during periods of intense neuronal activity, thereby supporting sustained neurotransmission and presynaptic membrane homeostasis [105, 106, 107, 108, 109].

#### 1.3.8 MEND (Massive Endocytosis)

MEND is triggered by acute cellular stressors that rapidly perturb PM homeostasis, including intracellular Ca^2+^ overload, hyperactivation of PKCθ/δ signaling, and transient increases in PI3Kγ activity [110, 111, 112]. This process proceeds independently of clathrin, dynamin and actin, as it is preferentially engaged in actin‐depleted regions of the PM [113]. The lack of actin requirement is a notable feature of this mechanism, as compared to other CIE routes, and reflects its reliance on large‐scale biophysical membrane reorganization triggered by a membrane phase separation process, promoted by palmitoylated proteins. Molecularly, DHHC5 (ZDHHC5) and its substrate acyl‐CoA induce protein palmitoylation cycles anchoring proteins at the PM, which promote endocytic initiation [112]. Also, cholesterol and PIP_2_ can independently contribute to the rapid induction of MEND within minutes following acute Ca^2+^ elevation, highlighting the cooperative role of lipid composition and signaling in driving membrane internalization [110, 111, 114]. The functional output of this pathway is to rapidly dissipate potentially pathological microclusters and reinstate membrane equilibrium to internalize ordered PM domains [110, 111, 112, 113, 114].

#### 1.3.9 UFE (Ultrafast Endocytosis)

UFE occurs at presynaptic active zones and retrieves ~60–80 nm synaptic vesicles within 100 ms of stimulation, matching the exocytosed membrane volume. In UFE, the reduction in membrane tension due to exocytic membrane addition primes the pathway, while subsequent Ca^2+^ influx induces the recruitment of endophilin, synaptojanin, dynamin and epsins [115], and stimulates local actin polymerization to drive rapid curvature and budding [116, 117]. Mechanistically, endophilin and synaptojanin promote rapid pit maturation and neck constriction, epsins contribute to membrane curvature generation, dynamin drives membrane fission, and actin provides the mechanical force required for fast invagination and vesicle scission throughout the process [115].

Collectively, these mechanisms underscore the diversity of CIE routes in neuronal cells, highlighting their adaptability in response to physiological stimuli ranging from synaptic activity to cellular stress.

#### 1.3.10 Macropinocytosis

Macropinocytosis mediates receptor‐independent bulk uptake of extracellular fluid and PM through large‐scale membrane ruffling. It is typically stimulated by RAS/PI3K‐dependent signaling pathways that promote actin remodeling at the PM. Functionally, macropinocytosis supports nutrient scavenging and pathogen surveillance and is capable of sustaining high endocytic flux. Its molecular machinery is highly adaptable, displaying overlapping cargo selectivity and interchangeable regulatory modules depending on cellular context [118, 119]. The resulting macropinosomes are large (0.2–5 μm), phase‐bright vacuoles formed by the closure of PM ruffles in a process dependent on Na+/H+ exchanger (NHE1) activity.

In mammalian cells, macropinocytosis exhibits pronounced cell‐type‐specific diversity in its molecular machinery, trafficking fate (degradation vs. recycling), and regulatory control. Macrophages and immature dendritic cells display constitutive membrane ruffling to support efficient antigen sampling and MHC loading, whereas fibroblasts and epithelial cells show minimal basal activity under steady‐state conditions but rapidly induce robust ruffle formation and macropinosome uptake in response to growth factor stimulation (e.g., PDGF or EGF) [120].

Under pathological conditions such as cancer (particularly in tumors harboring oncogenic RAS mutations) and during certain bacterial infections, macropinocytosis is markedly upregulated. In epithelial tumor cells, this pathway supports metabolic reprogramming by scavenging exogenous amino acids (e.g., glutamine), lipids, and proteins, thereby sustaining bioenergetic demands, proliferation, and immune evasion within nutrient‐poor microenvironments [120].

Thus, while mechanistically distinct from the “tubular” CIE mechanisms described above, macropinocytosis shares their actin dependence and clathrin independence and represents a high‐capacity and highly adaptable arm of the CIE network.

In conclusion, although multiple CIE routes have been described, they are unlikely to represent strictly segregated pathways. Rather, they appear to constitute partially overlapping, adaptable, and context‐dependent variations of shared membrane remodeling mechanisms. Consistent with this view, as discussed throughout this chapter, these processes rely on several common lipid and molecular determinants, including: (i) Nanoscale lipid organization (e.g., membrane rafts), (ii) actin cytoskeleton dynamics, (iii) membrane curvature regulators (e.g., BAR domain proteins, galectins, epsins), and (iv) cargo‐specific modifications (e.g., glycosylation, ubiquitination). Collectively, these shared features suggest that the boundaries between distinct CIE routes are dynamic and highly context‐dependent, reflecting a continuum of interconnected endocytic mechanisms rather than rigidly defined pathways.

### 1.4 Experimental Approaches to Study CIE: Integration of Genetic and Chemical Inhibitors

Experimental dissection of endocytic mechanisms is complicated by extensive overlap in the molecular machinery (e.g., adaptor proteins, lipids, regulatory factors, and cargoes), as well as by the compensatory activation of pathways in response to the chronic inhibition of another route. Such compensation frequently results in the adaptive rerouting of cargo trafficking [15, 121]. Consequently, selective inhibition of individual endocytic mechanisms, whether chemical or genetic, remains challenging and can confound data interpretation [76, 78].

Among the most widely used inhibitors are Dynasore and its second‐generation derivatives [122, 123, 124]. These compounds act as non‐competitive inhibitors of the GTPase activity of dynamins, thereby preventing dynamin‐mediated membrane scission while still permitting coat assembly and pit invagination. Consequently, these inhibitors have been instrumental in the study of CME and CIE [122, 123]. However, Dynasore and related compounds exhibit notable off‐target effects, including perturbation of actin polymerization even in dynamin triple‐knockout cells, with consequent effects on phagocytosis and macropinocytosis [125].

Some inhibitors are designed to disrupt protein–protein interactions rather than enzymatic activities, often necessitating higher concentrations to achieve biological efficacy. For example, the multivalent peptides D44‐ and D14‐Dyn2 were engineered to mimic dynamin's ability to engage multiple SH3 domains simultaneously, thereby inhibiting CME with micromolar affinity. Notably, this affinity can increase to the nanomolar range in the presence of both dimeric and tetrameric SH3 domains [126]. Pitstop 2 was developed to inhibit CME by blocking protein binding to the C‐terminal domain of clathrin, but its specificity has been questioned due to reported off‐target effects on CIE mechanisms [127, 128]. Continued efforts to refine such compounds and to develop new inhibitors with improved selectivity are ongoing. A case in point is Pitstop 2c/2d, which displays reduced cytotoxicity and improved affinity for clathrin, displacing adaptor proteins, such as epsins, from clathrin‐coated pits without measurably affecting CIE [129, 130].

Given the extensive cross‐reactivity of chemical inhibitors, rigorous validation using complementary approaches is essential. These include: (i) The use of pathway‐enriched markers or cargoes, whose selectivity is often cell‐type dependent; (ii) genetic perturbations using siRNA or CRISPR‐Cas9 to target specific endocytic components; and (iii) the expression of dominant‐negative constructs to achieve more acute pathway inhibition. A frequently overlooked caveat of genetic depletion strategies is that many endocytic proteins are highly stable, necessitating prolonged depletion times that may themselves trigger compensatory adaptations. Moreover, although dominant‐negative approaches allow more rapid inhibition, they can also be associated with cytotoxicity or neomorphic effects.

Table 1 summarizes commonly used chemical inhibitors employed to dissect endocytic pathways.

**TABLE 1:** Overview of drugs and small‐molecule inhibitors used to dissect CME and CIE pathways. The table summarizes the molecular targets, mechanisms of action, specificity, toxicity, and endocytic routes affected by the indicated compounds. Representative studies are included for each inhibitor. Notably, all chemical inhibitors exhibit some degree of toxicity and off‐target activity, with effects that depend on dose, treatment duration, and cellular context. Therefore, pharmacological inhibition alone is generally insufficient to unequivocally define a specific endocytic pathway and should be complemented by orthogonal approaches such as imaging, genetic ablation, and cargo‐tracking assays.

Target	Drug name	Mechanism	Dosage/unspecific effects	Toxicity	Endocytic route
CHC	Bolinaquinone [131]	Binding to the N‐terminal domain of CHC	Tested in silico [132]	Cytotoxic above ~6 μM [133]	CME
	Pitstop 1–2 [134, 135]		30 μM/can affect CIE [127, 128]	Cytotoxic above 30 μM [136, 137]	
	Pitstop 2c/2d [129]		~13–30 μM/only dextran (10 kDa) uptake tested (not affected) [129]	Cytotoxic above 100 μM [129]	
	ES9‐17 [138]		30 μM/not reported [138]	Cytotoxic above 30 μM [138]	
AP2/SNX9	Wbox2 [121]	Cell‐permeant peptide from N‐terminal domain of CHC	20 μM/can affect CIE [121]	Cytotoxic above 40 μM [121]	
Dynamin	Dynasore [122, 139]	Binding to the GTPase domain of DYN	~80–100 μM/affects dextran (10 kDa) uptake in dynamin TKO cells [41, 125]	Cytotoxic above 40 μM [140, 141]	CME FEME EGFR‐NCE
	Dyngo‐4a [123, 142]		~10–30 μM/affects dextran (10 kDa) uptake in dynamin TKO cells [123, 125, 142]	Minimal cytotoxicity [123, 142]	
	Dynole 34–2 [142, 143] Dynole 2–24 [142, 144]		~2–5 μM/not reported [143, 144]	Cytotoxic above 5 μM [124, 143, 144, 145]	
	Dynamin IN‐2 (compound‐43) [146]		10 μM/not reported [146]	Not reported	
	MiTMAB [147, 148]	Inhibits interaction with phospholipids	30 μM/not reported [148, 149]	Cytotoxic above 10 μM [145, 150, 151]	
Actin	Latrunculin [152]	Sequesters Actin monomers	Below 5 μM [78, 83, 153, 154, 155]/CME unaffected in the basolateral side of polarized cells [156]	Not reported/only short‐term treatment [78, 83, 153, 154, 155]	Most endocytic pathways
	Cytochalasin D [152, 157]	Caps Actin filaments leading to depolymerization	~5–6 μM/not reported [152, 154, 157, 158]	Not reported/only short‐term treatment [154, 158, 159]	
Cholesterol	Filipin [160]	Binds and sequesters cholesterol	~0.5–12 μg/mL/CME unaffected [39, 158, 161, 162]	Not reported/only short‐term treatment [39, 161, 162]	CLIC/GEEC EGFR‐NCE
	MβCD [163]	Disrupts lipid rafts and caveolae	~2–10 mM/CME affected [158, 163, 164]	Cytotoxic above ~10 mM [164, 165, 166]	CLIC/GEEC Macropinocytosis
Unknown	Ikarugamycin [167]	Disrupts clathrin‐coated pit formation and maturation	~2–4 μM/CIE unaffected [167, 168]	Cytotoxic above 4 μM [167, 168]	CME
Unknown	Chlorpromazine [169]	Disrupts clathrin assembly	~2–12 μM/can affect CIE [165, 169]	Cytotoxic above ~10 μM [165, 169]	CME
GBF1	LG186 [170]	Reversible Inhibitors of the ARF1 GEF GBF1	10 μM/CME unaffected [78, 171]	Not reported/only short‐term treatment [78, 171]	CLIC/GEEC
	AN‐96 [172]		25 μM/CME unaffected [172]	Minimal cytotoxicity [172]	
PICK1	FSC231 [173]	Binds to the PDZ domain of PICK1	~50–100 μM/CME unaffected [78]	Not reported	
CDC42	ML141 [174]	Inhibits the interaction of GTP‐bound CDC42 with effectors	10 μM/CME unaffected [171]	Cytotoxic above 10 μM [172, 175]	CLIC/GEEC FEME Macropinocytosis
Arp2/3	CK666 [176, 177, 178]	Disrupts Actin polymerization by stabilizing the inactive form of the Arp2/3 complex	50 μM/CME unaffected [44, 78]	Not reported/only short‐term treatment [179]	CLIC/GEEC, EGFR‐NCE Macropinocytosis
Dynein	Ciliobrevin D [180]	Blocker of the AAA+ ATPase motor	50 μM/CME slightly affected [44, 86, 181]	Cytotoxic above 200 μM [182]	CLIC/GEEC GL‐Lect FEME EGFR‐NCE
	Dynapyrazole A [182]	Blocks the microtubule binding site of dynein	15 μM/CME slightly affected [181, 182]	Cytotoxic above 20 μM [182]	
Unknown	7‐Ketocholesterol [183, 184]	Intercalates into the PM and inhibits tubulation	30 μM/CME unaffected [71, 185, 186]	Cytotoxic above 30 mM [71, 185]	CLIC/GEEC Macropinocytosis
Gal3	I3 [187]	Bind to carbohydrate recognition domains of Galectins, disrupting interaction with glycans	10 μM/CME unaffected [86, 188, 189]	Minimal cytotoxicity [168, 189, 190]	GL‐Lect
Pan‐Galectins	Lactose [191, 192, 193]		~100–200 mM/CME unaffected [83, 168, 189, 194]	Cytotoxic above 100 mM [189]	
GCS	GENZ‐123346 [195]	Inhibitors of glycosphingolipid biosynthesis	~2–5 μM in vitro; 0.225% (wt/wt) in vivo/CME unaffected [86, 188, 189, 196]	Minimal toxicity [189, 196]	
	PPMP [197]		5 μM/CME unaffected [83, 198]	Cytotoxic above 5 μM [83, 198]	
NHE1	EIPA [199, 200]	Inhibits Na + channels and Na+/H+ exchange	~25–75 μM/not reported [38, 201, 202, 203, 204]	Minimal cytotoxicity [203, 204]	Macropinocytosis
Unknown	Imipramine [205]	Blocks macropinosomes by inhibiting PM ruffling	10 μM/CME unaffected [205]	Minimal cytotoxicity [205]	
Unknown	Virapinib [206]	Unknown	~10–25 μM/not reported [206]	Minimal cytotoxicity [206]	

Abbreviations: CHC, clathrin heavy chain; Gal3, galectin‐3; GCS, glucosylceramide synthase; MβCD, methyl‐β‐cyclodextrin; PM, plasma membrane; Tf, transferrin.

### 1.5 Cargoes and Biological Functions of CIE

Over the past two decades, advances in imaging, molecular perturbation strategies, and quantitative trafficking assays have greatly expanded our understanding of cargoes internalized via CIE. A key concept emerging from these studies is that CME and CIE are often not mutually exclusive. Rather, they frequently internalize overlapping sets of cargoes, with the relative contribution of each mechanism depending on cell type, growth conditions, and signaling context [14]. As discussed above, unlike CME, which operates constitutively in most cells, CIE mechanisms are highly context‐dependent and are selectively engaged under specific physiological or pathological conditions [32]. Importantly, the endocytic route used by a given cargo strongly influences its intracellular fate, as different mechanisms preferentially direct internalized proteins toward recycling, retrograde trafficking, or lysosomal degradation. Consequently, cellular behaviors such as proliferation, the establishment of polarity, migration, and invasion are critically shaped by which endocytic pathways are active in a given context.

**FIGURE 2:**
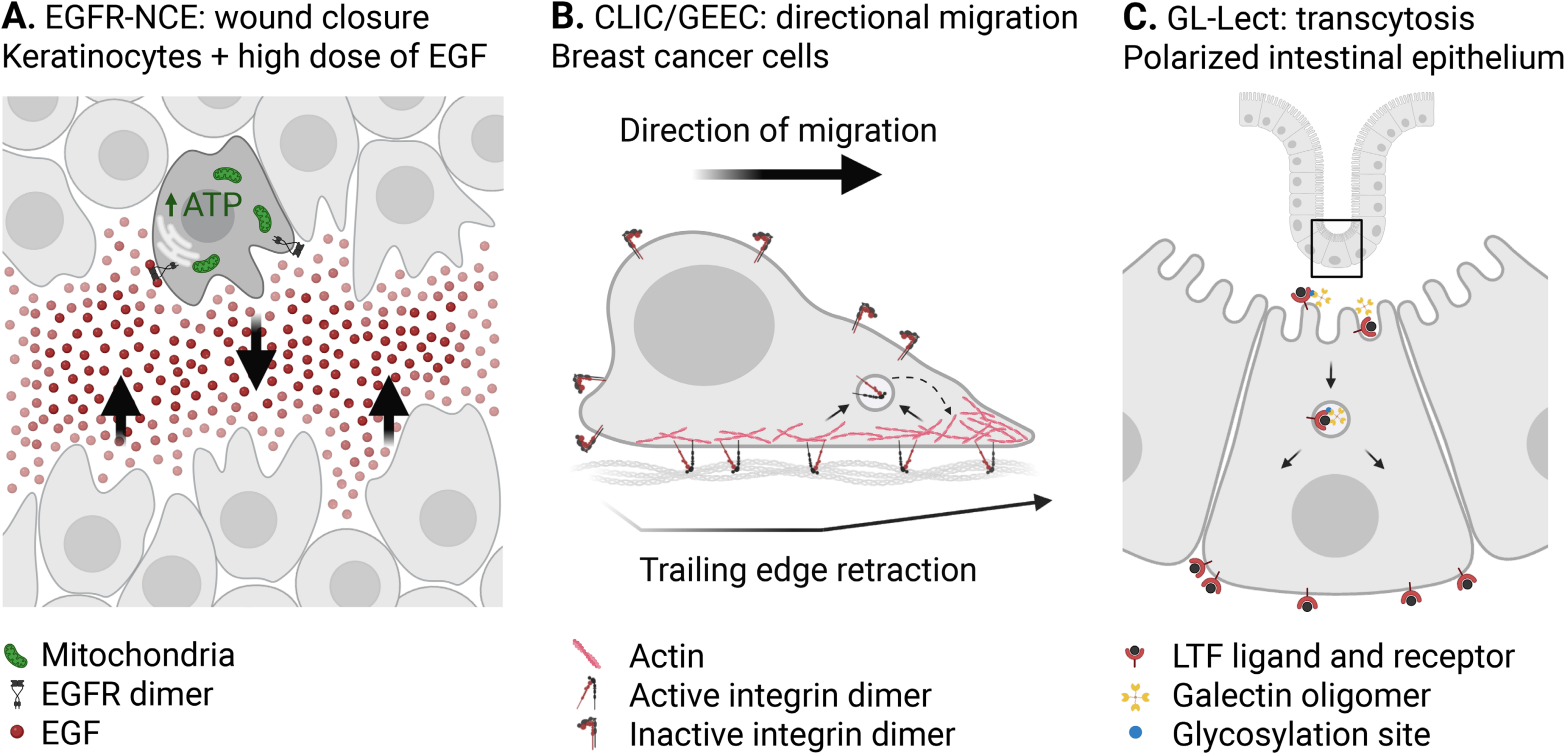
CIE role in physio‐pathological contexts. (A) During keratinocyte collective migration and wound closure under high EGF concentrations, EGFR non‐clathrin endocytosis (EGFR‐NCE) is activated. This mechanism couples receptor internalization to the formation of a PM‐ER‐mitochondria platform that enhances peripheral mitochondrial metabolism and local ATP production required for collective motility and efficient wound closure. (B) In migrating breast cancer cells, clathrin‐ and dynamin‐independent CLIC/GEEC (CG) endocytosis mediates the internalization of active integrin dimers from the basal cell surface and their recycling to the leading edge and/or to nascent focal adhesions. This process supports directional migration by coordinating Actin‐rich adhesion dynamics and trailing‐edge retraction, including the turnover of integrin‐based adhesions. (C) In polarized intestinal epithelium, the GL‐Lect mechanism drives apical‐to‐basolateral transcytosis. Extracellular galectin‐3 oligomers bind glycosylated cargo (e.g., lactotransferrin) and glycolipids at the apical surface, promoting cargo clustering, internalization into CLIC‐like carriers, and subsequent transcytotic trafficking across the intestinal epithelium. Created with Biorender.com.

A clear illustration of this principle is provided by EGFR endocytosis. At low ligand concentrations, EGFR is primarily internalized via CME, whereas at high, saturating doses of ligand, EGFR‐NCE is activated [39]. These two routes differentially regulate receptor fate: CME largely favors receptor recycling back to the PM, while EGFR‐NCE preferentially targets receptors for lysosomal degradation [92]. This observation led to the proposal that EGFR‐NCE functions as a protective, potentially tumor‐suppressive mechanism that limits excessive RTK signaling. However, EGFR‐NCE has also been linked to the enhanced migratory capacity of keratinocytes through the formation of PM‐ER‐mitochondria contact sites that locally stimulate ATP production required for cortical actin remodeling [44] (Figure 2A). Thus, although EGFR‐NCE attenuates receptor signaling by promoting degradation, it can simultaneously sustain spatially restricted signaling outputs that favor cytoskeletal remodeling and directional movement. This duality provides the cell with a coherent output at different ligand doses: at low doses, sustained signaling via CME promotes proliferation; at high doses, NCE activation inhibits proliferation while promoting migration [44]. Notably, the keratinocyte wound‐healing model currently represents the most compelling physiological evidence for EGFR‐NCE function in migration. Future studies employing genetic in vivo models and investigations in tumor settings will be required to establish the broader physiological and pathological relevance of this pathway. Particularly, how CIE controls growth factor responses in cancer remains an important open question. In breast cancer, RAB5A overexpression upregulates EGFR internalization via NCE and alters receptor sorting from endosomes. Consequently, active EGFR accumulates in endosomes, promoting sustained endosomal signaling, which supports collective cell motility and invasion [207, 208].

Consistent with a role in migration, CIE mechanisms such as FEME are strongly activated at the leading edge of migrating epithelial cells and primary dermal human fibroblasts [38]. In these regions, growth factor receptors such as EGFR are rapidly internalized, enabling dynamic receptor turnover and spatially confined signaling that drives directional movement. Endophilin A2 plays a central role in this process by promoting the formation and scission of tubular FEME carriers [90]. Through this activity, endophilin A2 couples local endocytosis to actin dynamics and signal transduction, thereby supporting cell motility and invasive behavior [88].

CIE pathways also regulate polarity and migration through the trafficking of adhesion molecules. The CLIC/GEEC and GL‐Lect mechanisms strongly influence PM organization by controlling the polarized distribution of glycoproteins and GPI‐anchored proteins. Following entry into early endosomes, these cargoes can either be recycled back to the PM or routed to the Golgi apparatus via retrograde trafficking before being delivered to specific PM domains. In particular, the CLIC/GEEC mechanism favors the endocytosis of active β1‐integrin from the basal surface of migrating cells, enabling its trafficking to endosomes and subsequent recycling to the leading edge and/or to nascent focal adhesions (Figure 2B) [81]. This spatially controlled recycling is essential for directional migration and efficient adhesion turnover in different breast cancer cells.

Notably, inactive β1‐integrin is internalized via GL‐Lect–driven endocytosis from the apical PM. This mechanism has been characterized in spontaneously migrating retinal pigment epithelial cells and in HeLa cells [83, 86]. Current data suggest a preferential association of bent‐closed integrins with GL‐Lect‐mediated uptake, whereas active integrins seem to be internalized preferentially via CLIC/GEEC and CME [86]. However, further studies across diverse cellular systems will be required to determine whether this reflects a general principle of integrin trafficking.

Interestingly, GL‐Lect triggered endocytosis is regulated by growth factor signaling through changes in cell surface glycosylation, as shown by evidence that EGF stimulation activates the neuraminidases NEU1 and NEU3, leading to desialylation of cell surface glycoproteins [85]. This “desialylation glycoswitch” enhances galectin binding and promotes GL‐Lect driven endocytosis of α3β1‐integrin, glycosphingolipids and additional cargoes, such as CD98. These cargoes are subsequently routed through the Golgi for resialylation and redistributed to the leading edge of migrating cells [85]. By dynamically remodeling adhesion molecules and membrane lipid organization, this mechanism can cooperate with growth factor signaling to promote invasive and migratory behavior in cancer cells.

A role for GL‐Lect endocytosis in epithelial physiology has been described in the transcytosis of lactotransferrin (LTF) across the intestinal epithelium (Figure 2C). In enterocytes, LTF binds to specific glycosylated receptors at the apical surface (e.g., Intelectin‐1), where galectin‐dependent lattice formation promotes its internalization via a GL‐Lect driven mechanism. Following endocytosis, LTF‐containing carriers are delivered to the basolateral membrane, thereby enabling transport across the epithelial barrier. This mechanism highlights how GL‐Lect endocytosis can support apico‐basal cargo delivery, extending the functional scope of CIE to epithelial transport and barrier functions (Figure 2C) [168].

Recent studies have further identified specialized CIE routes centered on endophilin A3 and extracellular lectins that selectively regulate adhesion molecules in cancer cells. An endophilin A3/galectin‐8‐dependent mechanism controls the internalization of the cancer‐associated adhesion molecule CD166, thereby influencing tumor cell adhesion and migration [84]. Similarly, immunoglobulin‐like adhesion molecules such as ICAM, L1CAM, and ALCAM are internalized via a GL‐Lect mechanism in an endophilin A3‐dependent manner [84, 209]. Their polarized redistribution following endocytosis contributes to immune synapse formation between cancer cells and CD8^+^ T lymphocytes, suggesting that CIE pathways also shape tumor–immune interactions and may be exploitable for therapeutic targeting [210].

Together, these observations underscore the biological relevance of CIE pathways and highlight their central roles in coordinating signaling, adhesion, and migration in both physiological and disease contexts.

### 1.6 Outlook: Lessons From the Past

The functional divergence between CME and CIE offers a useful lens on cellular complexity. If CME is a specialized machine and CIE a flexible toolkit, CME's stereotyped design illustrates a trade‐off between efficiency and adaptability. CME internalizes selected cargoes efficiently, but is less able than CIE to accommodate rapid fluctuations in membrane tension or large‐scale remodeling. Thus, the emergence and diversification of endocytic pathways, especially CIE routes, may illuminate early cellular organization and dynamics.

Compared with prokaryotes, eukaryotic cells have an elaborate, membrane‐bound internal organization. This reflects innovations, such as the nucleus, an extensive endomembrane system, a dynamic cytoskeleton, and mitochondria, which arose from an ancestral alphaproteobacterial endosymbiont [211, 212]. Endocytosis and the endomembranes likely marked a defining threshold in eukaryogenesis by overcoming diffusion constraints. Mitochondria supported this transition through the “ATP‐per‐gene” advantage, allowing genomic expansion and helping offset the metabolic cost of trafficking [213]. Tracing these pathways is therefore central to understanding how Eukarya emerged.

Comparisons among extant eukaryotes are only moderately informative, as most core trafficking components are conserved across major lineages [214]. Both CME [214] and CIE [75] are widespread, and current consensus holds that the Last Eukaryotic Common Ancestor (LECA) likely had a broad trafficking repertoire [215]. The key events therefore might have predated the LECA.

Endosymbiosis was central to eukaryotic origins: an alphaproteobacterial ancestor became a permanent resident in a host cell and evolved into mitochondria. Although the host identity was long debated, phylogenetic evidence now places eukaryotes firmly within the *Asgardarchaeota* superphylum (hereafter *Asgard*) [216, 217], within [216, 217, 218] or as sister to [219] the *Heimdallarchaeota*. *Asgard* genomes encode many eukaryotic signature proteins (ESPs) [220] once considered eukaryote‐specific. These include cytoskeletal components (actin homologues and gelsolin‐domain proteins), membrane‐remodeling systems (ESCRT complexes), and small GTPases (Gtr/Rag and Arf families) involved in trafficking, signaling, and nucleocytoplasmic transport [221, 222, 223]. Arf GTPases are especially relevant because they initiate inward membrane budding. These asgardian orthologs display functional similarities to their eukaryotic counterparts [222, 223, 224, 225, 226, 227, 228, 229, 230], providing an evolutionary blueprint for the emergence of eukaryotic internal architecture.

A central question is whether archaea, especially *Asgard*, possess an endomembrane system. Cultivation of *Candidatus Prometheoarchaeum syntrophicum* and *Candidatus Lokiarchaeum ossiferum* provided the first morphological insights, but neither species showed clear internal compartmentalization [229, 231, 232]. Both display actin‐rich protrusions, supporting the hypothesis that such structures promote interactions with, and possibly engulfment of, bacterial partners during eukaryogenesis [229].

The apparent absence of endomembranes in these organisms may reflect placement within *Lokiarchaeota*, which are more distant from eukaryotes than *Heimdallarchaeota*. Preliminary bioRxiv data suggest a more complex picture [233]. In that study, the Heimdallarchaeon *Candidatus Yibarchaeum umbracryptum* (*Ca. Y. umbracryptum*) displayed actin‐rich protrusions early in culture but later showed fewer protrusions and numerous intracellular vesicles. Cells also wrapped extracellular material, including other organisms, consistent with primitive endocytic‐like activity [233]. If confirmed, these observations suggest that a dynamic endomembrane system may have predated mitochondrial endosymbiosis. *Ca. Y. umbracryptum* lacks canonical eukaryotic trafficking homologs (coat proteins or ESCRT complexes) present in other *Asgard* lineages. Instead, its small genome encodes expanded small GTPases and SPFH‐family proteins, homologous to eukaryotic flotillins. Since flotillins participate in CIE in eukaryotes, these related proteins may drive membrane remodeling in *Ca. Y. umbracryptum*. If confirmed, this would support a model in which the earliest endocytic processes relied on CIE‐like mechanisms.

A recent large‐scale phylogenomic analysis further supports a dominant *Asgard* contribution to eukaryogenesis, identifying *Asgard* archaea as the main source of conserved eukaryotic functional systems, including an endomembrane apparatus [234]. In contrast, the alphaproteobacterial contribution appears restricted to energy metabolism and Fe–S cluster biogenesis. Genes from other bacterial phyla are present in eukaryotic genomes, but sporadically and without a consistent phylogenetic pattern [234]. These findings favor a model of eukaryogenesis in which the foundational eukaryotic organization emerged within the *Asgard* lineage leading to the LECA. It was then expanded by the alphaproteobacterial endosymbiont and further shaped by intermittent horizontal gene transfers (HGT) from diverse bacteria.

Alternative models invoke “three or more‐partners” [211, 235, 236]. Some propose that an *Asgard* archaeon was first internalized by a bacterial host, with loss of the original archaeal membrane before a second endosymbiosis produced mitochondria. Such models address a longstanding problem: The chemical and enzymatic incompatibility between archaeal and bacterial membrane lipids [237]. Because eukaryotic membranes contain bacterial‐type lipids, archaeal‐host models must explain both this transition and its selective advantage.

A preliminary bioRxiv study [238] may offer clues. Using different criteria for core pangenome reconstruction compared with previous studies [234], the authors inferred a larger bacterial and viral contribution to eukaryogenesis. Beyond alphaproteobacteria, major contributions were attributed to *Planctomycetota, Chloroflexota, Myxococcota*, and *Nucleocytoviricota* viruses [238]. If confirmed, these findings would fit a pre‐LECA evolutionary landscape shaped by multiple symbiosis and extensive HGT. Temporal modeling suggests early interactions between an *Asgard* archaeon and *Planctomycetota*, followed by additional bacterial contributions and an intermediate or late acquisition of mitochondria. Late acquisition of mitochondria is also supported by analysis of eukaryotic gene sequences using a relaxed molecular clock methodology [239]. The *Planctomycetota* contribution is enriched in lipid metabolism genes, potentially explaining the bacterial‐like lipid composition of eukaryotic membranes [237]. *Planctomycetota* also have unusual traits, including the presence of coat‐like proteins [240] and phagocytosis‐like prey engulfment [241]. These features make them candidates for a primordial host in a bacterial‐archaeal fusion, although this hypothesis remains debated [242, 243, 244].

Overall, eukaryogenesis, including the emergence of the endomembrane system, remains an evolving paradigm. Even so, growing phylogenomic data and quantitative models (see, for example, REF [245]) are moving the field from conceptual frameworks towards empirically grounded reconstructions. These lessons from the past may deepen our understanding of membrane trafficking and may inform future strategies to manipulate these processes for fundamental and applied purposes.

## 2 Concluding Remarks

In conclusion, the functional significance of CIE extends beyond cargo internalization per se. Different CIE routes selectively channel cargoes toward distinct intracellular destinations—including recycling, retrograde transport, transcytosis, or degradation—thereby influencing signaling, polarity, migration, and epithelial barrier functions. This coupling of uptake mechanism to cargo fate represents a central principle underlying the functional specialization of CIE. Viewed through an evolutionary lens, the diversification of CIE pathways may reflect the progressive adaptation of an ancestral membrane‐remodeling toolkit to the increasingly complex trafficking and signaling demands of eukaryotic cells.

## Funding

This work was supported by European Research Council, ERC‐CoG2020 101002280, Fondazione AIRC per la ricerca sul cancro ETS, IG 24415, IG 23060, Ministero dell’Università e della Ricerca (MUR), 2022W93FTW_002, EU‐CN00000041, the Italian Ministry of Health, Ricerca Corrente 2023‐2024 and 5 per 1000 funds, RF‐2021‐12373957, PNRR‐MCNT2‐2023‐12378490, and Worldwide Cancer Research, 26‐0124.

## Conflicts of Interest

The authors declare no conflicts of interest.

## Data Availability

Data sharing not applicable to this article as no datasets were generated or analysed during the current study.
